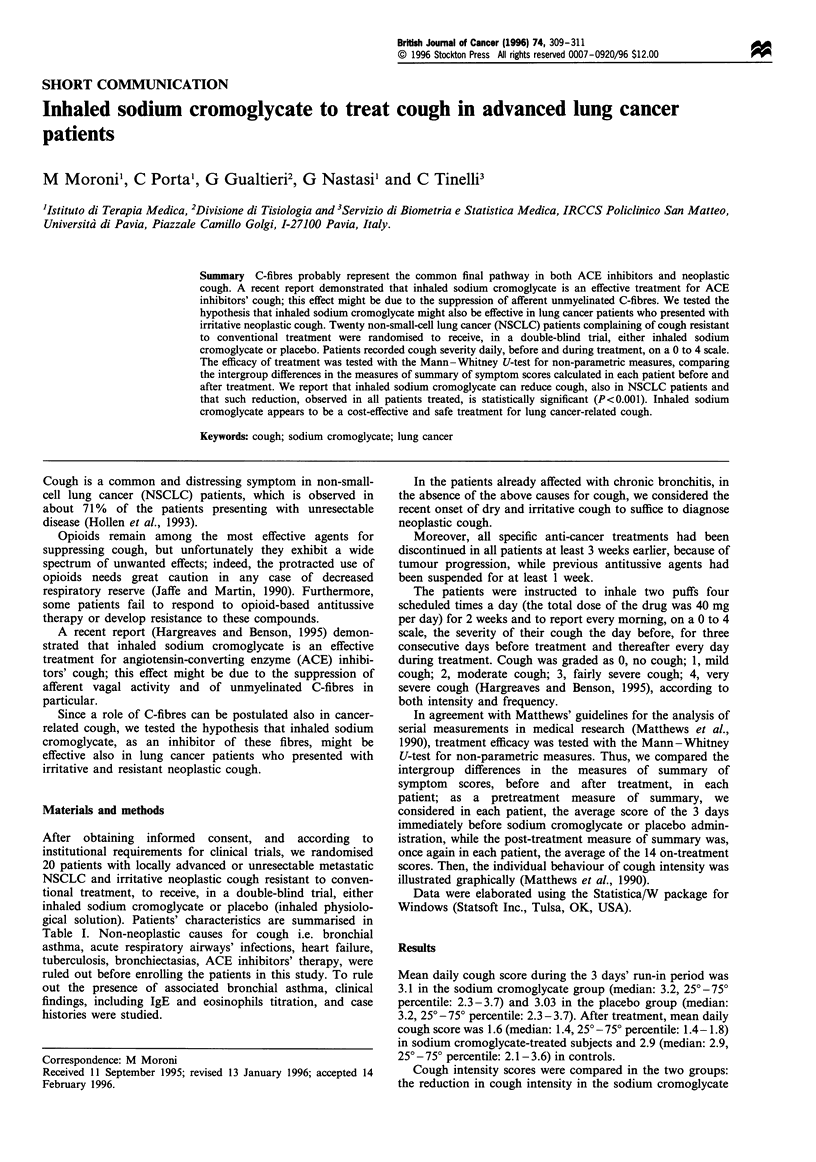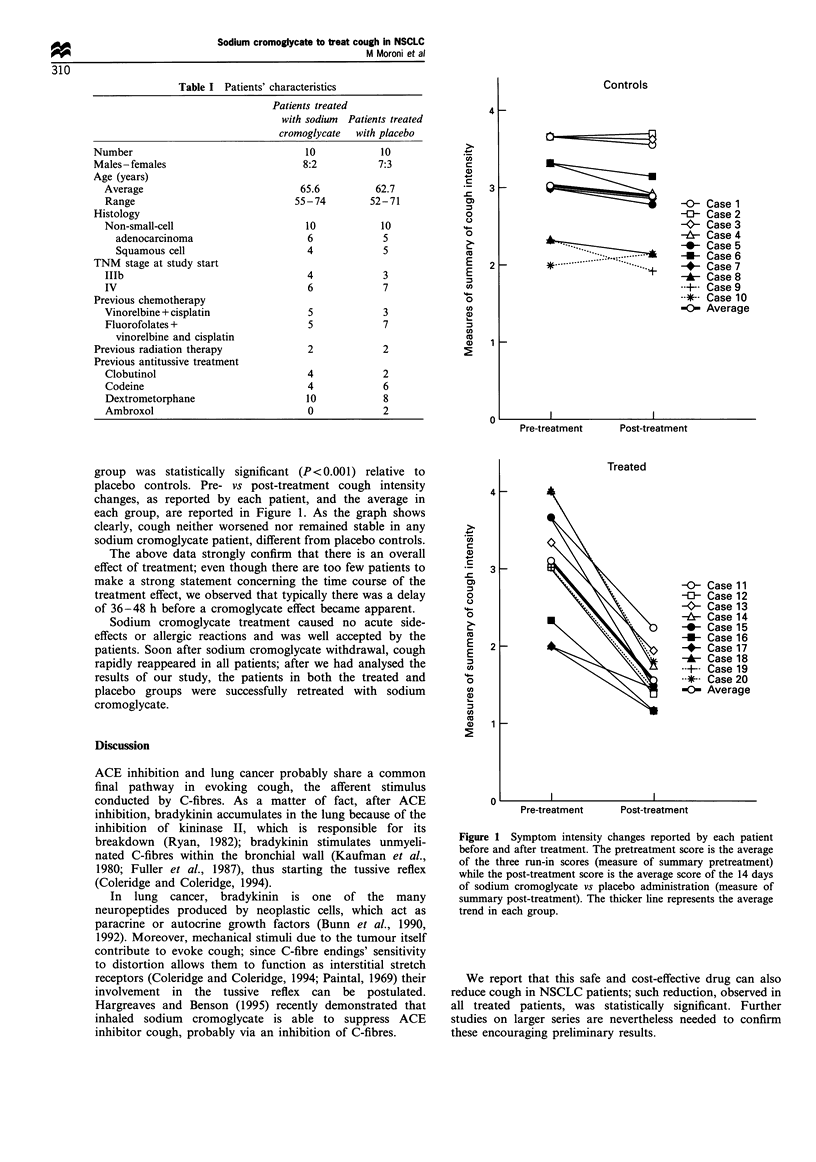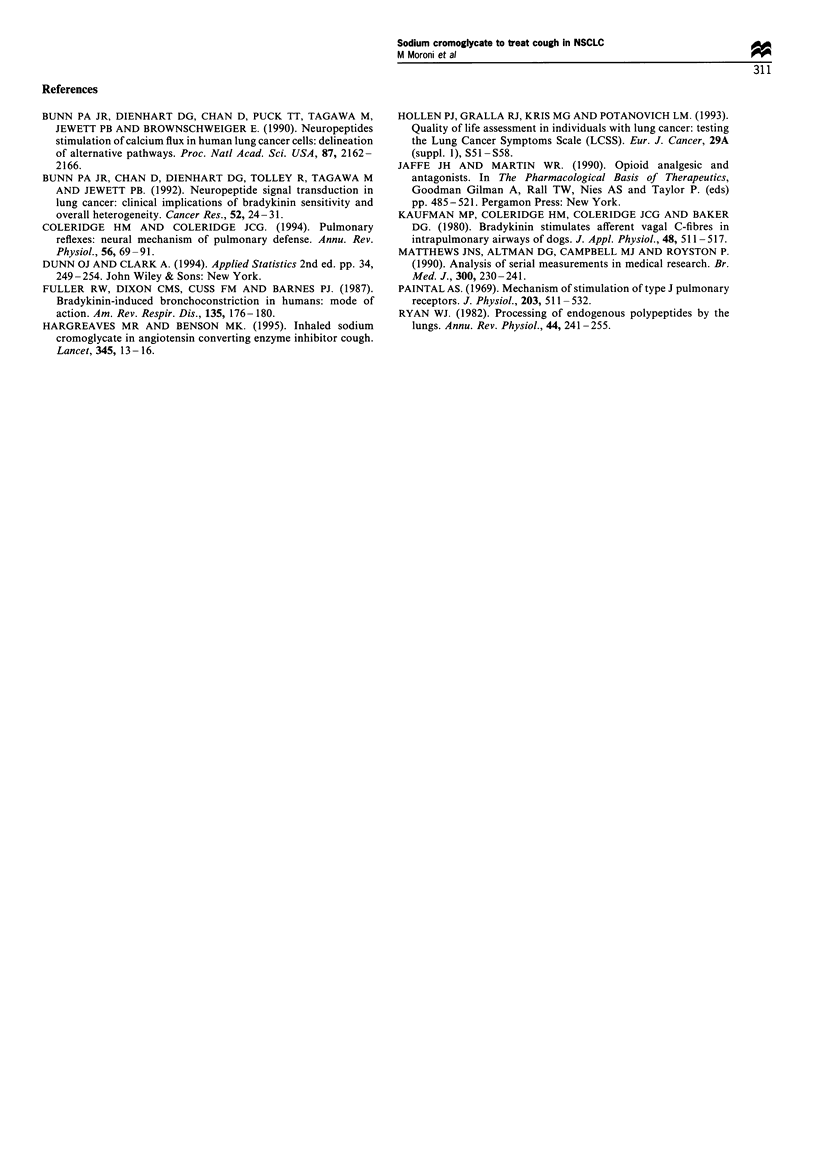# Inhaled sodium cromoglycate to treat cough in advanced lung cancer patients.

**DOI:** 10.1038/bjc.1996.358

**Published:** 1996-07

**Authors:** M. Moroni, C. Porta, G. Gualtieri, G. Nastasi, C. Tinelli

**Affiliations:** Istituto di Terapia Medica, IRCCS Policlinico San Matteo, Università di Pavia, Italy.

## Abstract

C-fibres probably represent the common final pathway in both ACE inhibitors and neoplastic cough. A recent report demonstrated that inhaled sodium cromoglycate is an effective treatment for ACE inhibitors' cough; this effect might be due to the suppression of afferent unmyelinated C-fibres. We tested the hypothesis that inhaled sodium cromoglycate might also be effective in lung cancer patients who presented with irritative neoplastic cough. Twenty non-small-cell lung cancer (NSCLC) patients complaining of cough resistant to conventional treatment were randomised to receive, in a double-blind trial, either inhaled sodium cromoglycate or placebo. Patients recorded cough severity daily, before and during treatment, on a 0 to 4 scale. The efficacy of treatment was tested with the Mann-Whitney U-test for non-parametric measures, comparing the intergroup differences in the measures of summary of symptom scores calculated in each patient before and after treatment. We report that inhaled sodium cromoglycate can reduce cough, also in NSCLC patients and that such reduction, observed in all patients treated, is statistically significant (P < 0.001). Inhaled sodium cromoglycate appears to be a cost-effective and safe treatment for lung cancer-related cough.


British Journal of Cancer (1996) 74, 309-311
© 1996 Stockton Press All rights reserved 0007-0920/96 $12.00

SHORT COMMUNICATION

### Inhaled sodium cromoglycate to treat cough in advanced lung cancer patients

M Moroni[1], C Porta[1], G Gualtieri[2], G Nastasi[1] and C Tinelli[3]

[1]*Istituto di Terapia Medica,* [2]*Divisione di Tisiologia and* [3]*Servizio di Biometria e Statistica Medica, IRCCS Policlinico San Matteo, Università di Pavia, Piazzale Camillo Golgi, I-27100 Pavia, Italy.*

**Summary** C-fibres probably represent the common final pathway in both ACE inhibitors and neoplastic cough. A recent report demonstrated that inhaled sodium cromoglycate is an effective treatment for ACE inhibitors' cough; this effect might be due to the suppression of afferent unmyelinated C-fibres. We tested the hypothesis that inhaled sodium cromoglycate might also be effective in lung cancer patients who presented with irritative neoplastic cough. Twenty non-small-cell lung cancer (NSCLC) patients complaining of cough resistant to conventional treatment were randomised to receive, in a double-blind trial, either inhaled sodium cromoglycate or placebo. Patients recorded cough severity daily, before and during treatment, on a 0 to 4 scale. The efficacy of treatment was tested with the Mann-Whitney *U*-test for non-parametric measures, comparing the intergroup differences in the measures of summary of symptom scores calculated in each patient before and after treatment. We report that inhaled sodium cromoglycate can reduce cough, also in NSCLC patients and that such reduction, observed in all patients treated, is statistically significant ($P<0.001$). Inhaled sodium cromoglycate appears to be a cost-effective and safe treatment for lung cancer-related cough.

**Keywords:** cough; sodium cromoglycate; lung cancer

Cough is a common and distressing symptom in non-small-cell lung cancer (NSCLC) patients, which is observed in about 71% of the patients presenting with unresectable disease (Hollen *et al.*, 1993).

Opioids remain among the most effective agents for suppressing cough, but unfortunately they exhibit a wide spectrum of unwanted effects; indeed, the protracted use of opioids needs great caution in any case of decreased respiratory reserve (Jaffe and Martin, 1990). Furthermore, some patients fail to respond to opioid-based antitussive therapy or develop resistance to these compounds.

A recent report (Hargreaves and Benson, 1995) demonstrated that inhaled sodium cromoglycate is an effective treatment for angiotensin-converting enzyme (ACE) inhibitors' cough; this effect might be due to the suppression of afferent vagal activity and of unmyelinated C-fibres in particular.

Since a role of C-fibres can be postulated also in cancer-related cough, we tested the hypothesis that inhaled sodium cromoglycate, as an inhibitor of these fibres, might be effective also in lung cancer patients who presented with irritative and resistant neoplastic cough.

### Materials and methods

After obtaining informed consent, and according to institutional requirements for clinical trials, we randomised 20 patients with locally advanced or unresectable metastatic NSCLC and irritative neoplastic cough resistant to conventional treatment, to receive, in a double-blind trial, either inhaled sodium cromoglycate or placebo (inhaled physiological solution). Patients' characteristics are summarised in Table I. Non-neoplastic causes for cough i.e. bronchial asthma, acute respiratory airways' infections, heart failure, tuberculosis, bronchiectasias, ACE inhibitors' therapy, were ruled out before enrolling the patients in this study. To rule out the presence of associated bronchial asthma, clinical findings, including IgE and eosinophils titration, and case histories were studied.

In the patients already affected with chronic bronchitis, in the absence of the above causes for cough, we considered the recent onset of dry and irritative cough to suffice to diagnose neoplastic cough.

Moreover, all specific anti-cancer treatments had been discontinued in all patients at least 3 weeks earlier, because of tumour progression, while previous antitussive agents had been suspended for at least 1 week.

The patients were instructed to inhale two puffs four scheduled times a day (the total dose of the drug was 40 mg per day) for 2 weeks and to report every morning, on a 0 to 4 scale, the severity of their cough the day before, for three consecutive days before treatment and thereafter every day during treatment. Cough was graded as 0, no cough; 1, mild cough; 2, moderate cough; 3, fairly severe cough; 4, very severe cough (Hargreaves and Benson, 1995), according to both intensity and frequency.

In agreement with Matthews' guidelines for the analysis of serial measurements in medical research (Matthews *et al.*, 1990), treatment efficacy was tested with the Mann-Whitney *U*-test for non-parametric measures. Thus, we compared the intergroup differences in the measures of summary of symptom scores, before and after treatment, in each patient; as a pretreatment measure of summary, we considered in each patient, the average score of the 3 days immediately before sodium cromoglycate or placebo administration, while the post-treatment measure of summary was, once again in each patient, the average of the 14 on-treatment scores. Then, the individual behaviour of cough intensity was illustrated graphically (Matthews *et al.*, 1990).

Data were elaborated using the Statistica/W package for Windows (Statsoft Inc., Tulsa, OK, USA).

### Results

Mean daily cough score during the 3 days' run-in period was 3.1 in the sodium cromoglycate group (median: 3.2, 25°-75° percentile: 2.3-3.7) and 3.03 in the placebo group (median: 3.2, 25°-75° percentile: 2.3-3.7). After treatment, mean daily cough score was 1.6 (median: 1.4, 25°-75° percentile: 1.4-1.8) in sodium cromoglycate-treated subjects and 2.9 (median: 2.9, 25°-75° percentile: 2.1-3.6) in controls.

Cough intensity scores were compared in the two groups: the reduction in cough intensity in the sodium cromoglycate

Correspondence: M Moroni
Received 11 September 1995; revised 13 January 1996; accepted 14 February 1996.

**Table I** Patients' characteristics

| | *Patients treated with sodium cromoglycate* | *Patients treated with placebo* |
|---|---|---|
| Number | 10 | 10 |
| Males–females | 8:2 | 7:3 |
| Age (years) | | |
| Average | 65.6 | 62.7 |
| Range | 55–74 | 52–71 |
| Histology | | |
| Non-small-cell | 10 | 10 |
| adenocarcinoma | 6 | 5 |
| Squamous cell | 4 | 5 |
| TNM stage at study start | | |
| IIIb | 4 | 3 |
| IV | 6 | 7 |
| Previous chemotherapy | | |
| Vinorelbine + cisplatin | 5 | 3 |
| Fluorofolates + vinorelbine and cisplatin | 5 | 7 |
| Previous radiation therapy | 2 | 2 |
| Previous antitussive treatment | | |
| Clobutinol | 4 | 2 |
| Codeine | 4 | 6 |
| Dextrometorphane | 10 | 8 |
| Ambroxol | 0 | 2 |

group was statistically significant ($P<0.001$) relative to placebo controls. Pre- *vs* post-treatment cough intensity changes, as reported by each patient, and the average in each group, are reported in Figure 1. As the graph shows clearly, cough neither worsened nor remained stable in any sodium cromoglycate patient, different from placebo controls.

The above data strongly confirm that there is an overall effect of treatment; even though there are too few patients to make a strong statement concerning the time course of the treatment effect, we observed that typically there was a delay of 36–48 h before a cromoglycate effect became apparent.

Sodium cromoglycate treatment caused no acute side-effects or allergic reactions and was well accepted by the patients. Soon after sodium cromoglycate withdrawal, cough rapidly reappeared in all patients; after we had analysed the results of our study, the patients in both the treated and placebo groups were successfully retreated with sodium cromoglycate.

### Discussion

ACE inhibition and lung cancer probably share a common final pathway in evoking cough, the afferent stimulus conducted by C-fibres. As a matter of fact, after ACE inhibition, bradykinin accumulates in the lung because of the inhibition of kininase II, which is responsible for its breakdown (Ryan, 1982); bradykinin stimulates unmyelinated C-fibres within the bronchial wall (Kaufman *et al.*, 1980; Fuller *et al.*, 1987), thus starting the tussive reflex (Coleridge and Coleridge, 1994).

In lung cancer, bradykinin is one of the many neuropeptides produced by neoplastic cells, which act as paracrine or autocrine growth factors (Bunn *et al.*, 1990, 1992). Moreover, mechanical stimuli due to the tumour itself contribute to evoke cough; since C-fibre endings' sensitivity to distortion allows them to function as interstitial stretch receptors (Coleridge and Coleridge, 1994; Paintal, 1969) their involvement in the tussive reflex can be postulated. Hargreaves and Benson (1995) recently demonstrated that inhaled sodium cromoglycate is able to suppress ACE inhibitor cough, probably via an inhibition of C-fibres.

**Figure 1** Symptom intensity changes reported by each patient before and after treatment. The pretreatment score is the average of the three run-in scores (measure of summary pretreatment) while the post-treatment score is the average score of the 14 days of sodium cromoglycate *vs* placebo administration (measure of summary post-treatment). The thicker line represents the average trend in each group.

We report that this safe and cost-effective drug can also reduce cough in NSCLC patients; such reduction, observed in all treated patients, was statistically significant. Further studies on larger series are nevertheless needed to confirm these encouraging preliminary results.

### References

BUNN PA JR, DIENHART DG, CHAN D, PUCK TT, TAGAWA M, JEWETT PB AND BROWNSCHWEIGER E. (1990). Neuropeptides stimulation of calcium flux in human lung cancer cells: delineation of alternative pathways. *Proc. Natl Acad. Sci. USA*, **87**, 2162–2166.

BUNN PA JR, CHAN D, DIENHART DG, TOLLEY R, TAGAWA M AND JEWETT PB. (1992). Neuropeptide signal transduction in lung cancer: clinical implications of bradykinin sensitivity and overall heterogeneity. *Cancer Res.*, **52**, 24–31.

COLERIDGE HM AND COLERIDGE JCG. (1994). Pulmonary reflexes: neural mechanism of pulmonary defense. *Annu. Rev. Physiol.*, **56**, 69–91.

DUNN OJ AND CLARK A. (1994). *Applied Statistics* 2nd ed. pp. 34, 249–254. John Wiley & Sons: New York.

FULLER RW, DIXON CMS, CUSS FM AND BARNES PJ. (1987). Bradykinin-induced bronchoconstriction in humans: mode of action. *Am. Rev. Respir. Dis.*, **135**, 176–180.

HARGREAVES MR AND BENSON MK. (1995). Inhaled sodium cromoglycate in angiotensin converting enzyme inhibitor cough. *Lancet*, **345**, 13–16.

HOLLEN PJ, GRALLA RJ, KRIS MG AND POTANOVICH LM. (1993). Quality of life assessment in individuals with lung cancer: testing the Lung Cancer Symptoms Scale (LCSS). *Eur. J. Cancer*, **29A** (suppl. 1), S51–S58.

JAFFE JH AND MARTIN WR. (1990). Opioid analgesic and antagonists. In *The Pharmacological Basis of Therapeutics*, Goodman Gilman A, Rall TW, Nies AS and Taylor P. (eds) pp. 485–521. Pergamon Press: New York.

KAUFMAN MP, COLERIDGE HM, COLERIDGE JCG AND BAKER DG. (1980). Bradykinin stimulates afferent vagal C-fibres in intrapulmonary airways of dogs. *J. Appl. Physiol.*, **48**, 511–517.

MATTHEWS JNS, ALTMAN DG, CAMPBELL MJ AND ROYSTON P. (1990). Analysis of serial measurements in medical research. *Br. Med. J.*, **300**, 230–241.

PAINTAL AS. (1969). Mechanism of stimulation of type J pulmonary receptors. *J. Physiol.*, **203**, 511–532.

RYAN WJ. (1982). Processing of endogenous polypeptides by the lungs. *Annu. Rev. Physiol.*, **44**, 241–255.